# The impact of buying intention of global fashion on local substitute: The role of product design and price

**DOI:** 10.1016/j.heliyon.2023.e22160

**Published:** 2023-11-10

**Authors:** Sri Zuliarni, Dwi Kartikasari, Bambang Hendrawan, Siti Sri Windrayati Siregar

**Affiliations:** Business and Management, Politeknik Negeri Batam, Batam, Indonesia

**Keywords:** Global, Local, Fashion, Substitute, Design, Price

## Abstract

In a competitive environment, the relationship between global and local fashion in shaping consumption behavior is under question. This study explores the antecedents of consumers' purchasing intentions for local fashions, mainly considering buying intentions for global alternatives, price, and product design while controlling for age, income, gender, geographic location, and occupation. We discovered that consumers’ buying intentions for global fashion harm their intent to buy local substitutes, based on a non-probability survey of 260 young adults and hierarchical multiple regression analysis. We associate these findings with cultural identity theory, social identity theory, ethnocentrism, substitute versus complementary concepts, and cue utilization theory. International marketers can devise marketing strategies referring to these findings.

## 1 Introduction

Fashion is one of the most globalized industries worldwide, frequently leading a country down the road of export-oriented industrial growth. Its low entry barriers, such as minimal setup fees and small upfront investment, contribute to the creation of billions of employment. The gear industries add value by creating jobs, improving the economy, transferring expertise, and broadening the market. The world hegemony of fashion brands has been held by renowned enterprises from the United States of America (USA), Japan, and Europe since the introduction of international procurement in the 1980s [1].

In 2021, global exports of clothing manufactured goods increased by 19.6%. Despite the pandemic, textile exports retain a high value and significant

Growth. Global trade is worth more than one trillion dollars, with USA, Japan, and the United Kingdom being the topmost importers. Although the European Union exports and imports, its trade balance in the clothes industry is negative. For the past two decades, China, Bangladesh, Vietnam, Indonesia, Malaysia, Turkey, and India have been among the top ten exporters [2].

Fashion is derived from the Latin *facere*, which translates to create or do. According to European cultural histories, “fashion” is a dual process of producing and doing, participating in cultural practices such as a look, a garment, or even societal differences between factions of individuals [3]. Fashion includes clothing and accessories created by designers who wish to represent their personalities and cultures to themselves and their customer base [4]. In this paper, we use the term “global fashion” to refer to fashions imported from foreign countries worldwide. Fashions produced by national manufacturers for domestic markets are called “local substitutes” for global fashion.

The pervasiveness of smartphones since the COVID crisis has stimulated the rise of globalization to a whole new level via cross-border e-commerce. The online platform enables global fashion to easily and quickly penetrate domestic markets [5]. To help local manufacturers compete with the global giants, many studies and our study attempt to explain the antecedents of consumers’ purchasing intentions for specifically local products. Buying intention of global fashion, product design, and price are antecedents we are particularly interested in because we underlie our approach to address the research problems on existing popular theories and concepts, including a) cultural identity theory, b) social identity theory, c) substitute and complementary concepts, and d) cue utilization theory.

Our work is important because, one, it elevates the study of the relationship between global fashion and local alternatives through a causal analysis approach. Most studies separately focus on international fashion [6] or domestic fashion [7]. Some works discuss concurrently, but most do so through a comparative analysis [8,9]. We argue that social identity theory validates competition between the in-group of local fashion and the out-group of imported apparel, which justifies the substitution effect in consumer shopping intent [10]. Our research fills a gap in the antecedents of domestic goods purchase intentions by incorporating the highly competitive environment they are currently facing and expects to add to the equation.

Two, we expand the global and local discourse by involving cue utilization. Numerous product cues have been examined in many studies to predict the purchase intention of domestic fashion [9]. We expect our findings to contribute to comparing the significant drivers from product cues in the marketing mix and from a competitive point of view. Three, we are focusing on young adults in a developing country—Indonesia, one of the world's largest textile exporters. Young adults are the biggest chunk of Indonesia's population. It is the fourth most populous nation globally, making it attractive for global fashion marketers. Understanding this vast segment of consumers will help managers devise their marketing strategies in entering a market dominated by local fashions.

We organize this paper with an introduction, a conceptual framework, a methodology, results, discussions, limitations, and conclusions. In the next section, we develop hypotheses based on our literature review. We then discuss our findings in detail in the results section. In the last section, we conclude and explain the limitations of this research that make room for future research.

## 2 Literature review

### 2.1 Cultural identity theory

Consumer culture theory investigates how customers consciously modify and reshape symbolic value embedded in brands, marketing, or products to materialize their specific individual and societal contexts and, after that, their lifestyle and identity aspirations [11]. It establishes a theoretical basis for the premise of cultural identity theory that purchasers have generalizable attitudes toward global and local products. Cultural identity theory has been used to explain the relationship between consumers’ attitudes toward global and local merchandise [12].

Customers can have a negative attitude toward global products while being positive toward local substitutes. This pairing of attitudes can be classified as “local”. Because consumers embrace home-country consumer culture, they favor local consumption selections and enjoy stronger (presumed) uniqueness [13].

Nevertheless, numerous internationalization scholars have emphasized that considerable segments of consumers have favorable feelings toward global and local products. The global and the local interact, resulting in distinctive effects in each time and place, referred to as “*glocal*” affiliations for several young consumers. *Glocal* buyers would like to mix local and global products in their consumption playlists innovatively. Consumers in India [14], Brazil, and Russia [15] strongly prefer local and global brands.

### 2.2 Substitute and complementary concepts

The interaction between preferences for global and local products can be damaging, meaning that an increase (decrease) in preferences for global products affects a decrease (increase) in local alternatives. This interaction is called a substitute. However, the interplay can be positive where an increase (decrease) of preferences for global affect an increase (decrease) for local alternatives. This interaction is called complementary [16]. The substitute effect occurs for global or local identity consumers, whereas the complementary effect occurs for *glocal* identity consumers [17].

### 2.3 Social identity theory

This study also draws upon social identity theory that explains intergroup prejudice. It presents the idea of the in-group (us, local) and out-group (them, foreign) and asserts that in-group members have the propensity to support their in-group over the out-group. The exposure of an out-group can elicit an intergroup conflicting, competitive, or discriminatory reaction to the in-group [18]. This theory is among the most influential principles used to explain the ethnocentrism phenomenon when consumers favor local over imported products [19]. The above three ideas from social identity theory, cultural identity theory, and substitute and complementary concepts underlie the competitive effect of global fashion on local substitutes.

### 2.4 Purchase intentions

Buying intention is the openly expressed or aware behavioral intent or proposal to purchase. Commonly speaking, intention becomes synonymous with behavior [10]. We use buying intention rather than actual purchase because it is one of the most popular topics for marketers to predict the shopping intent of domestic products [20]. Its analysis can determine the success or failure of customer satisfaction with a particular service or product, which will ultimately contribute to its actual sale [21]. Second, actual purchases of foreign or domestic products have been empirically tricky to predict because consumers may not tell their actual purchase [22], or circumstances may place them in a position to purchase something other than what they intended to due to availability, impulse buying, and so on [23].

### 2.5 Cue utilization theory

Cue utilization theory contends that the motivational composition of action constantly acts to achieve the optimal range of cue utilization and thus justifies several experimental steps to provide a concise interpretation of a collection of information about conscientious behavior [24]. In our study, the latter means the behavior of purchasing local fashions. The product choice cues can be intrinsic, related to physical attributes of local fashion like product design, or extrinsic, related to non-physical attributes of fashion like price and origin (home country or foreign). Intrinsic cues cannot be amended without manipulating the fashion's characteristics [25]. Extrinsic cues are broader in scope, hence relatable to a greater array of products, but they are subject to sample variation like age, gender, etc. [26].

We select only two cues, product design representing intrinsic cues and price from extrinsic cues because these two are significant drivers for buying domestic apparel for fashion leaders and fashion followers [9]. Price and product design are essential elements of marketing mixes that marketers usually pay attention to sell their products. Although other studies might enlist a handful of cues to understand fashions, we believe that irrelevant cues might hinder the optimal range of cue utilization. We use gender, age, occupation, rural, and income as control variables to ensure the comprehensiveness of our analysis.

## 3 Conceptual framework

### 3.1 Model 1: The effect of shoppers’ demographic background on buying intention of local fashion

Demographics have long been covariates in abundant marketing studies. Age, gender, education, and income are most commonly used. Age is frequently found to be significant [26,27]. In global local discourse, young adults are open to debate [15]. In the fashion industry, young adults [7] are also more sensitive to product design and price, which makes them a justified sample for our study. However, we still segment the age of young adults from 15 to 40 years and correlate these data as confounding variables to our independent variable of purchasing intent for domestic fashion.

Although less often than age, the level of income is also found to contribute to the local-global discourse via ethnocentrism [27]. The higher the consumers’ income, the higher their social status, and the more ethnocentric they are, the more likely they are to purchase domestic products [28].

Gender is not commonly found to be a significant contributing factor in global-local discourse [15,27]. But women are usually more sensitive to fashion than men, so some fashion studies focus only on females [9]. Geographic location and occupation are also among less major covariates than age, but we include them anyway to limit the influence of these control variables in our model. The location of our samples may influence our observed variable; rural consumers may be less exposed to global fashion than urban consumers and thus prefer buying local rather than global. Regarding occupation, the unemployed might be more sensitive to price when buying fashion than people with jobs. Hence, we develop the following hypothesis:

H1 Shoppers' demographic background (age, income, gender, geographic location, and occupation) influence their buying intention of local fashion

**Fig. 1:**
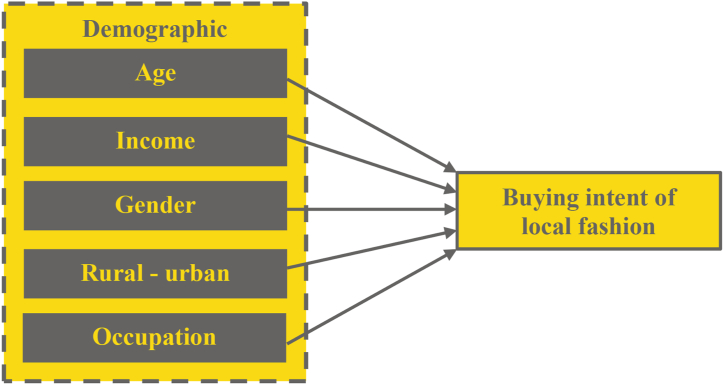
Model 1 The effect of demographic to buying intention of local fashion.

We do not state the positive or negative sign of the above hypothesis because the control variables are nominal or ordinal data. Hence, we only want to know their significance in the equation, not their signs. Fig. 1 describes the hypothesis as Model 1 as follows:

We put model 1 in equation (1):

(1) $$Y_{1}=a+B_{1}\;\left(X_{1}\right)+B_{2}\;\left(X_{2}\right)+B_{3}\;\left(X_{3}\right)+B_{4}\;\left(X_{4}\right)+B_{5}\;\left(X_{5}\right)+E$$

Y_1_ = buying intention of local fashion for Model 1

a = constant

B_1-5_ = coefficients of demographic variables

X_1-5_ = age, income, gender, geographic location, and occupation

E = model error

### 3.2 Model 2: The effect of buying intention of global fashion to local substitute

In a competitive environment, consumers' intentions toward global fashion can influence their intentions toward local fashion. Reciprocally, the consumers’ buying intentions for local fashion can influence their intentions for global fashion. Competition for similar brands or products works in both directions and offers greater predictive power [10]. In this paper, we do not focus on disclosing the two-sided directions of the global-local relationship, but we focus on the buying intention of local fashion as our primary variable; hence, we offer the following hypothesis:

H2 When controlling for demographic, shoppers buying intentions of global fashion negatively influence their buying intentions of local alternatives

**Fig. 2:**
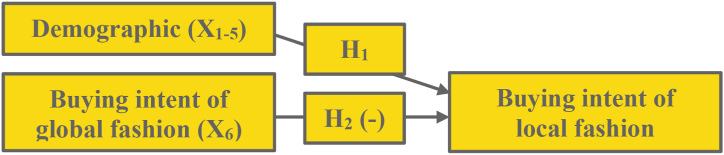
Model 2 The effect of buying intention of global fashion to buying intention of local alternatives controlling demographics.

We state the negative direction of the above hypothesis because a past study shows that competition hurts focal choice. After all, focal and alternative products usually act as substitutes, which is usually valid for consumers with local or global cultural identities. However, the negative direction might not apply to consumers with a glocal cultural identity who desire to combine both products in their consumption behavior. As a result, it is critical to examine the relationships between these two options to determine whether they are positive or negative in a developing-country setting. Fig. 2 describes the above hypothesis as Model 2 – as follows:

We put model 2 in equation (2):

(2) $$Y_{2}=a+B_{1‐5}\;\left(X_{1‐5}\right)\;–\;B_{6}\;\left(X_{6}\right)+E$$

Y_2_ = buying intention of local fashion for Model 2

B_6_ = coefficients of buying intention of global fashion

X_6_ = buying intention of global fashion

### 3.3 Model 3: The effect of price and product design on buying intention of local fashion

Price is the most commonly used variable among the 4Ps to estimate shopping behavior significantly [21,29]. Price sensitivity drives consumers’ preferences when buying products considering their foreign or local origins [30]. In the fashion industry, price is also a vital predictor [9,12,30,31]. In a developing country setting, where respondents might be more price sensitive than those in a developed country, price is a paramount predictor of buying intention [12,31,32].

In this paper, we ask about the affordability of locally-made fashion. Lower price in ordinal data means affordable. On the contrary, a high price denotes an expensive item. Price is a tricky attribute because, as an extrinsic cue, it can signal different things. When consumers associate price with quality, also called net utility [10], they might think the higher the price, the higher the quality, and the higher the consumers’ desire to buy. However, consumers can also associate price with value for money or worth. In this case, they can assume that the higher the price, the less value there is for the consumer, the less it is worth, and the fewer consumers desire to buy it. Thus, we do not hypothesize positive or negative signs for price.

Cultural identity theory mandates that customers relate their purchasing behavior with their cultural lifestyle identity [12,15]. Local fashion designs represent their home country's style [4,9]. The more unique and appealing the design is to domestic taste, the more consumers desire to buy local fashions [9]. Because product design is a straightforward intrinsic, we hypothesize a positive direction for design. We offer the following hypothesis:

H3 Price has an increment effect while product design has an increment positive effect on the negative relationship of shoppers buying intentions of global fashion and their buying intentions of local alternatives when controlling for demographic

**Fig. 3:**
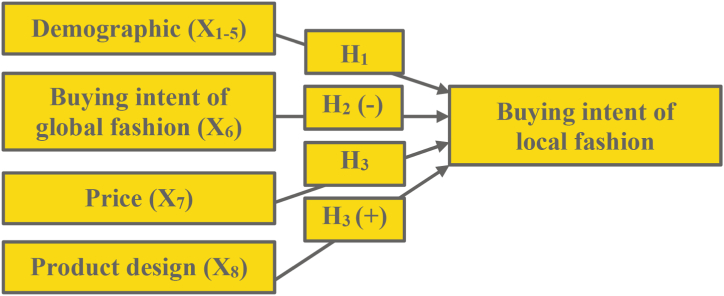
Model 3 The effect of buying intention of global fashion to buying intention of local alternatives adding price and product design controlling demographics.

Fig. 3describes the above hypothesis as Model 3 as follows:

We put model 3 in equation (3):

(3) $$Y_{3}=a+B_{1‐5}\;\left(X_{1‐5}\right)\;–\;B_{6}\;\left(X_{6}\right)+B_{7}\;\left(X_{7}\right)+B_{8}\;\left(X_{8}\right)+E$$

Y_3_ = buying intention of local fashion for Model 3

B_7,8_ = coefficients of price and product design

X_7,8_ = price and product design

## 4 Methodology

### 4.1 Data collection

We collected data using a self-administered online questionnaire. Compared to face-to-face or in-person surveys, this data collection method ensures greater efficiency and anonymity while reducing interviewer bias. However, the low monitoring ability of this method makes it difficult for respondents to ask questions. The author has subject-matter experts validated the instruments to address this issue [33]. The authors and two academics from the college separately reviewed the questionnaire before we spread it. Politeknik Negeri Batam ethically approves the questionnaire. At the beginning of the questionnaire, we confirmed all participants’ informed consent, confidentiality, and voluntary ethics. We introduced the definition of “fashion,” which includes clothing, shoes, and bags. And we explained the meaning of global and local fashion.

We surveyed the students in our college and asked them to broadcast the link to the questionnaire to their friends via Whatsapp social media. Hence, we implemented non-probability sampling by mixing two sampling modes: convenience and snowball. We limited our interference in the questionnaire filling because our study setting was non-contrived. Data collection was 30 days. We collected 413 responses, screened them, and finalized them with 260 complete data points.

### 4.2 Measure

Our questionnaire is in Bahasa Indonesia. Our constructs are single-item measures as we want our respondents to have a simple, quick, clear, but meaningful survey [34]. Purchase intention was sufficient with one indicator because it is unambiguous, narrow, and well-defined [35]. We use a 4-point Likert scale. We translate our wording and measurement to English for paper writing. For purchase intention, we stated, “I intend to buy global (or local) fashion.” For product design, “I would choose local fashion because of its stylish product design.” For the price, “the price of local fashion is affordable.” Demographics are straightforward.

### 4.3 Data analysis

We analyzed the data using hierarchical multiple regression analysis on SPSS software. We initially had a two-item scale for the purchase intention of global fashion, a two-item scale for the purchase intention of local fashion, and a five-item scale for product attributes, including price and product design. We choose the most valid and reliable item from these items for this research [36]. We keep the remaining items to test convergent validity, which results in all significant Pearson correlation at a minimum value of 0.625 for global fashion purchase intention (X_6_), indicating that all items are valid. Testing single-item reliability using parallel-form reliability is not explained in this limited-space paper [35].

We tested classical assumptions before proceeding with regression. We found that the variable of purchase intention in global fashion (X_6_) significantly meets the linearity test but not step model 3, X_7_ and X_8_. However, we do not plan to model the best type of regression equation. Thus, we can ignore the linearity test as it is less relevant to confirming our hypotheses. The model satisfies the normality test of residuals, as confirmed by the significant Mahalanobis distance. This model also satisfies the multicollinearity test, where the correlations between each independent variable are less than 0.8, with product design and price having the greatest value at 0.525. Their Std. Error and unstandardized B coefficients are less than 1, VIF less than 10 with product design at a maximum of 1.385, and tolerance more than 0.01 with product design at 0.722, confirming the absence of multicollinearity issues. The model satisfies negative and positive autocorrelation, with Durbin Watson at 1.946. The Park test does not detect the presence of heteroscedastic error when all calculated t is less than t_table_.

## 5 Results

### 5.1 Descriptive statistics

Our samples are skewed toward females (60.8 %), those who are 21–25 years old (53.5 %), students (73.5 %), low-income households (53.1 %), and those who live in urban areas (96.2 %), as shown in Table 1. The universities in Indonesia are usually established in urban areas. It is no surprise that our samples mostly live in urban areas. Although we do not limit our study to a specific gender, as conducted by past studies [9], our focus on fashion naturally attracts more women's samples than men. The skewness in our samples limits the generalizability of our findings to a different population profile.

**Table 1:** Samples descriptive statistics.

Gender	#	%
Female	158	60.8
Male	102	39.2
Age		
15–20	98	37.7
21–25	139	53.5
26–30	21	8.1
31–35	1	.4
35–40	1	.4
Occupation		
Student	191	73.5
Private employee	45	17.3
Public servant	4	1.5
Stay home mom	5	1.9
Unemployed	15	5.8
Income		
< Rp. 1.000.000	138	53.1
Rp. 1.000.000 – Rp.3.000.000	56	21.5
Rp.3.000.000 – Rp. 5.000.000	55	21.2
> Rp. 5.000.000	11	4.2
Geographic location		
Urban	250	96.2
Rural	10	3.8

The skewness in marketing literature is common as most studies utilize non-probability sampling [10,[37], [38], [39]]. Although studies have shown that student samples are adequate for many studies, using student samples was less desirable in our study because age has been shown to correlate with purchasing behavior [16,40] and thus may risk more potential bias than consumer samples [38].

We noticed that sample responses to all four variables range from 1 for strongly disagreeing to 4 for strongly agreeing. The average buying intention of global fashion is 2.77 lower than that of local fashion, which is 3.17, meaning that our samples prefer local fashion to global alternatives. However, global fashion fans vary more than local fashion fans as shown in Table 2 where global fashion buying intention is 0.786, higher than local fashion buying intention at 0.697. The skewness and kurtosis for all variables are close to zero, which signifies their symmetric and normal distribution. At the very least, the skewness is from −2 to 2, and the kurtosis is from −7 to 7 [41].

**Table 2:** Variables descriptive statistics.

Variables	Mean	Std. Deviation	Skewness	Kurtosis
Product design of local fashion	3.36	.614	−.500	−.085
Price of local fashion	3.41	.643	−.809	.433
Buying intention of global fashion	2.77	.786	−.191	−.389
Buying intention of local fashion	3.17	.697	−.513	.137

### 5.2 The empirical results

In model 1, we utilize the least squares method with the control variables (age, income, gender, geographic location, and occupation) as the first block, followed by the main effects (purchase intention of global fashion) in step 2, and the additional effects of price and product design in equation (3). We also looked at variance inflation factors (VIFs), as detailed in Table 3, to see if multicollinearity influenced the outcomes. As mentioned earlier in the methodology section, the VIFs were way less than 10 in each of the three steps of the model, indicating that multicollinearity did not affect the controls and causal variables.

**Table 3:** Hierarchial multiple regression analysis on buying intention of local fashion.

Predictor variables	B	t-value	p-value	Std Err	β-value	VIF
Step 1: control variables (df = 254)						
Constant	2.643***	8.934	.000	.296		
Gender	−.022	−.244	.807	.090	−.015	1.032
Age	.153**	2.095	.037	.073	.145	1.241
Occupation	−.008	−.176	.860	.048	−.013	1.363
Income	−.035	−.625	.532	.057	−.047	1.474
Rural - urban	.351	1.555	.121	.226	.097	1.014
Model 1 fitness	R^2^ = .027	F = 1.394	.227			
Model change	ΔR^2^ = .027	ΔF = 1.394	.227			
Step 2: main effects (df = 253)						
Constant	3.282***	10.288	.000	.319		
Gender	−.056	−.648	.518	.087	−.039	1.040
Age	.145**	2.063	.040	.071	.137	1.242
Occupation	−.015	−.314	.754	.047	−.022	1.365
Income	−.021	−.378	.706	.055	−.027	1.479
Rural - urban	.414*	1.902	.058	.218	.115	1.018
Global fashion's buy intent	−.239***	−4.478	.000	.053	−.269	1.015
Model 2 fitness	R^2^ = .098	F = 4.591	.000			
Model 2 change	ΔR^2^ = .071	ΔF = 20.053	.000			
Step 3: increment effects (df = 251)						
Constant	3.285***	7.658	.000	.429		
Gender	−.065	−.750	.454	.086	−.045	1.044
Age	.135*	1.901	.058	.071	.127	1.265
Occupation	−.000	.000	1.000	.047	.000	1.416
Income	−.029	−.529	.598	.054	−.038	1.486
Rural - urban	.448**	2.063	.040	.217	.124	1.023
Global fashion's buy intent	−.239***	−4.490	.000	.053	−.270	1.024
Product Design	.152*	1.911	.057	.080	.134	1.398
Price	−.154**	−1.985	.048	.078	−.142	1.452
Model 3 fitness	R^2^ = .116	F = 4.107	.000			
Model 3 change	ΔR^2^ = .018	ΔF = 2.495	.085			

*p < .1; **p < .05; ***p < .01; dependent variable: buying intention of local fashion.

In step 1, only age is positively associated with the purchase intention of local fashion (p-value = .037, B = 0.153). The R^2^ indicates that only 2.7% of the variance in the dependent variable can be explained by predictor variables, mainly age as a control variable (F(254) = 1.394, p-value = .227). Therefore, H_1_ is partially supported, meaning that shoppers’ demographic background, especially age, influences their buying intentions for local fashion. Ethnocentric tendencies drive the positive influence of age on local buying intentions. As consumers age, they are more inclined to be ethnocentric [27] and more likely to buy local products. We do not find support for past studies that back other demographic parameters such as income [27,28] and gender [9,27].

Next, in step 2, Table 3 shows that the main effect of global fashion's buying intention is undoubtedly significant. By adding the competition effect from global fashion, we can predict the purchase intention of local fashion from the insignificant simultaneous effect of model 1 (F(254) = 1.394, p-value = .227) to the simultaneous effect of model 2 (F(253) = 4.591, p-value = .000) with a rocketing change ΔF = 20.053. The R^2^ also rises from only 2.7% to 7.1%, meaning that the variance in the dependent variable can be explained by age and geographic location (rural–urban) as control variables and global fashion's buy intent, respectively (p-value = .040, B = 0.145; p-value = .058, B = 0.414; p-value = .000, B = −0.239). Thus, H_2_ is supported, meaning that when controlling for age and geographic location, shoppers buying intentions of global fashion negatively influence their buying intentions of local alternatives. This finding substantiates a competitive environment [10] that influences consumers' behavior towards substitutes [42].

Finally, adding the effects of product design and price on local fashion's purchasing intention is significant to the equation in step 3. By adding two cues from the marketing mix, i.e., product design and price, we can increase the predictability of the purchase intention of local fashion from model 2 (F(253) = 4.591, p-value = .000) to model 3 (F(251) = 4.107, p-value = .000) with a small change ΔF = 2.495. The R^2^ rises a little from only 7.1% to 11.6%, meaning that the variance in the dependent variable can be explained by age and geographic location (rural–urban) as control variables, global fashion's buy intent, product design, and price, respectively (p-value = .058, B = 0.135; p-value = .040, B = 0.448; p-value = .000, B = -0.239; p-value = .057, B = 0.152; p-value = .048, B = -0.154). As a result, H_3_ is supported, meaning that price has a significant negative increment effect. While some studies [50] contradict this finding, it is consistent with earlier studies [8], critically hails the price vs. quality trade-offs and rebuffs price-sensitivity in apparel shopping [43]. In contrast, product design has a significant positive increment effect to the negative relationship of shoppers buying intentions of global fashion and their buying intentions of local alternatives when controlling for age and geographic location.

## 6 Discussions, implications, and limitations

### 6.1 The effect of shoppers’ demographic background on buying intention of local fashion

Age is an influential predictor of buying intentions for local fashion. Our findings show a positive association, meaning that older consumers are likelier to buy local fashion than younger ones. However, we have to underline that the scope of our study is only for young adults aged 15–40. The phenomenon might be indirectly influenced by ethnocentrism, the tendency to prefer domestic to foreign goods, although many studies are ambiguous. The older the consumers, the more ethnocentrism they develop and the more likely they are to purchase a local product [27,44].

Another explanation could be that as consumers age, they become less aware of product offerings [45]. As a result, they are more likely to attach themselves to their convenient local culture, preferring local fashion. The younger generation is more aware of current global trends and emerging fashions [46]; they are more technologically active than the older age group and use e-commerce more often; they are open to global-local discourse [15]; hence, they are not attached too close to local cultural identity and local fashion purchasing behavior.

Rural-urban discourse, which was initially not found to be significant in Model 1, which focuses solely on local fashions, emerges significantly once global local discourse is introduced in Models 2 and 3. The positive sign in our study means that rural consumers, not urban ones, prefer local fashions. The geographic location where our respondents live might influence their variance in purchasing behavior. Rural consumers might be less exposed to global fashion than urban ones; hence, they express more intention to buy local than global. The heterogeneity of rural and urban consumers also appears consistently in past studies for other products, such as renewable energy [47,48]. Because urban consumers are more exposed to technologies and e-commerce [49], they are more likely than rural customers to buy global products via e-commerce.

Marketing managers selling global fashion should take these findings as an opportunity to market their products to younger consumers living in urban areas, as these consumers have yet to attach themselves to the local cultural identity too closely and might welcome global fashion more than older rural consumers. Our study also implies that managers selling local fashion should pay more attention to older adults and the rural group as they are more likely to buy local fashion.

This study limits itself to young adults in Indonesia. Different age groups and different countries might demonstrate further findings. Future research should expand the sample outreach to other age groups or countries.

### 6.2 The effect of buying intention of global fashion on local substitute

In a competitive environment, consumers’ intentions to buy global fashion negatively influence their intention to buy local fashion. Competition hurts local fashion because global and local fashion are substitutes [10]. Indonesian consumers exhibit local cultural identity where ethnocentrism is considered among the highest in the world compared to 139 other countries. This study confirms the preference for domestic products in Indonesia, Honduras, Vietnam, Nicaragua, El Salvador, Myanmar, Guatemala, and the Philippines [50].

Marketing managers selling global fashion should take these findings as an opportunity to market their products to new countries—119 countries showing more cosmopolitan and glocal cultural identity than the ones mentioned. Global fashion marketers will have a better chance of selling their products to consumers with glocal cultural identities who desire to combine both products in their consumption behavior.

Note that the just-mentioned ethnocentric countries like Indonesia and the Philippines have considerably big economies, with large and diversified domestic sectors at the time of this study. Global marketers wishing to enter these markets with intense local cultural identity should devise strategies considering local culture.

Our findings provide empirical evidence of competition's effect on the relationship between the buying intentions of global fashion and local fashion. The competition results from a strong local cultural identity, introduced by cultural identity theory, and ethnocentrism emerged from social identity theory. We also provide empirical evidence of global and local fashion's substitutional, not complementary, effects on purchasing behavior. Our findings support past studies that found that preferences for substitute products are consistently correlated [42] and extend the applicability of substitute, not complementary, concepts in explaining the purchase intention of domestic fashion.

This study limits its scope to the impact of the purchase intention of global fashion on that of a local substitute, not the other way around, that is, the impact of local fashion purchase intentions on global alternatives. Although competition should have worked in both directions [10], we cannot prove it in this paper and encourage future research to explore this area.

### 6.3 The effect of price and product design on buying intention of local fashion

We found that price affordability negatively influences buying intentions for local fashion. The more affordable the price set for local fashions, the less likely consumers will buy them. This finding is consistent with past studies [8], at the same time, contradictory to some other studies [51]. The cue utilization theory explains the reason for these incoherent findings of price variables is that price is an extrinsic cue subject to sample variation [26]. Consumers might utilize the cue of price in association with quality, also called net utility [10]; they might think the higher the price, the less affordable, and the higher the quality, the higher the consumers’ desire to buy. In this sense, price as an extrinsic cue has a more prevalent impact than price as an economic trade-off because classic price-demand equations dictate that the higher the price set for a product, the lower its demand and the less desirable it is to consumers.

The price premium consumers are willing to pay for local fashion might come from their attachment to fashion as their cultural and social identity; in the same manner, consumers are willing to pay a premium price for luxurious brands [52]. The relationship between the perception of price and quality and the buying intention of domestic apparel is also found in other settings, namely China [53]. In a more complex context, the less affordable the price of local fashions, the more likely they are worth showing off to conspicuous consumers, and the more unique they are, the more consumers want them.

Fashion design as an intrinsic cue works more straightforwardly than price. Consistent with past research, we found that the better the product design [4], the more unique it is, the more it represents consumers’ cultural and social identity, and the more likely they are to want it [9,53]. Cultural identity theory claims customers relate their purchasing behavior with their cultural lifestyle identity [12,15].

Our findings imply practical implications for local fashion manufacturers in Indonesia. They should not rope themselves into price wars. Instead, they should compete on new ideas for product design and enjoy price premiums. This strategy is appropriate for local fashion marketers because the Indonesian fashion market associates price with quality and, thus, is willing to pay a premium for good style and design. Other countries, like Thailand, might have different tendencies where their purchase intention of local fashion, in this case, jeans, is less than that of global fashion. Thus, local fashion manufacturers should discount their prices to compete [54].

Our study enriches the literature supporting extrinsic and intrinsic cues for cue utilization theory. We highlight different logical explanations behind these two cues. The extrinsic cue of price necessitates a more complex explanation to comprehend a signal and what it represents—quality. This logical explanation from a marketing perspective outperforms the classic price demand equilibrium. In comparison, the intrinsic cue is more straightforward.

However, we screen out the causality analysis from our study. Further studies can direct experiments to verify the causality of global-local consumer behavior. We neither cover specific fashion products in our study. Although we explained to the respondents that fashions include clothing, bags, and shoes, we question whether each product category or sub-product type, like shoes—sportswear, might exhibit different findings. Last, we do not cover the details of product design that consumers want. We leave those areas for future research.

## 7 Conclusions

In a competitive environment, consumers' intentions to buy global fashion negatively influence their intention to buy local fashion. Competition harms local fashion because global and local fashion are substitutes. By factoring in the intense competition, our research fills a gap in the antecedents of domestic product purchase intentions. We also found that price affordability negatively influences buying intentions for local fashion. Consumers may use price as a cue to quality: the higher the price, the less affordable; the higher the quality, the greater the consumers' desire to buy. Practitioners and marketers can revisit pricing strategies by referencing our findings overlooking price sensitivity in apparel shopping. The latter provides empirical support for cue utilization theory, which offers polarizing logical explanations behind extrinsic and intrinsic cues. Fashion design as an intrinsic cue works more straightforwardly than price. We found that the better the product design, the more unique it is, and the more it represents consumers' cultural and social identities, the more likely they want it. Last, we discovered that age shows a positive association, meaning that older consumers are more likely to buy local fashion than younger ones, and rural consumers prefer local fashion over urban customers. Our study's generalizability is limited to our sampling context: fashion, youth, e-commerce, and Indonesia's scope and methodology. Prospective research should expand the setting to include various samples, product categories, and countries. Further studies can direct experiments to verify the causality of global-local consumer behavior.

## Data availability statement

Data associated with this study been deposited into a publicly available repository https://doi.org/10.5281/zenodo.10065372

## CRediT authorship contribution statement

**Sri Zuliarni:** Conceptualization, Data curation, Formal analysis, Funding acquisition, Investigation, Methodology, Project administration, Resources, Software, Supervision, Validation, Visualization, Writing – original draft, Writing – review & editing. **Dwi Kartikasari:** Conceptualization, Data curation, Formal analysis, Funding acquisition, Investigation, Methodology, Project administration, Resources, Software, Supervision, Validation, Visualization, Writing – original draft, Writing – review & editing. **Bambang Hendrawan:** Conceptualization, Data curation, Funding acquisition, Project administration, Resources, Writing – original draft. **Siti Sri Windrayati Siregar:** Data curation, Formal analysis, Methodology, Resources, Writing – original draft.

## Declaration of competing interest

The authors declare the following financial interests/personal relationships which may be considered as potential competing interests:Dwi Kartikasari reports financial support was provided by Politeknik Negeri Batam. Dwi Kartikasari reports a relationship with Batam State Polytechnic that includes: employment.
