# Supplementary material for: The impact of buying intention of global fashion on local substitute: The role of product design and price
Source: Heliyon. 2023 Nov 10;9(11):e22160. doi: 10.1016/j.heliyon.2023.e22160 (PMC10689880; doi:10.1016/j.heliyon.2023.e22160)
Supplement: Multimedia component 1 [file mmc1.docx]

Notes: The actual questionnaire is in Bahasa Indonesia. This is the translated version in English.

Greetings. My name is xxx from Politeknik Negeri Batam. I am conducting a research using questionnaire as primary data collection. I humbly ask your favor to voluntarily spare less than 5 minutes to participate in our survey. Please fill in the following form completely and honestly. I will keep your confidentiality. Please contact me at +62xxx or [xxx@xxx.ac.id](mailto:xxx@xxx.ac.id) for further details. Thank you for your response!

Respondent’s section

Name :

Contact (Email or phone for clarification purposes, optional) :

1. Gender
2. Female
3. Male
4. Age :
5. 15-20 years c. 31-35 years
6. 21-25 years d. > 35 years
7. 26-30 years
8. Occupation :
9. Student d. Stay home mom
10. Private employee e. Unemployed
11. Civil servant f. Others……..
12. Income :
13. < Rp. 1.000.000
14. Rp. 1.000.000 – Rp.3.000.000
15. Rp.3.000.000 – Rp. 5.000.000
16. > Rp. 5.000.000
17. Geographic location :
18. Urban
19. Rural

Please circle/select the number below that represents how you feel for each statement

| Statements | Strongly agree | Somewhat agree | Somewhat disagree | Strongly disagree |
| --- | --- | --- | --- | --- |
| I intend to buy **global** fashion | 4 | 3 | 2 | 1 |
| I intend to buy **local** fashion | 4 | 3 | 2 | 1 |
| I would choose local fashion because of its stylish product design | 4 | 3 | 2 | 1 |
| The price of local fashion is affordable. | 4 | 3 | 2 | 1 |
| I am going to buy **global** fashion in the next 12 months | 4 | 3 | 2 | 1 |
| I am going to buy **local** fashion in the next 12 months | 4 | 3 | 2 | 1 |

Original questionnaire in Bahasa Indonesia

Perkenalkan, nama saya xxx dari Politeknik Negeri Batam, Jurusan Manajemen Bisnis. Penelitian ini menggunakan kuesioner sebagai alat pengumpulan data primer. Oleh karena itu, saya mohon bantuan teman-teman untuk berkenan meluangkan waktu kurang dari 5 menit untuk mengisi / memberikan jawaban atas terkait dengan penelitian ini. Saya mengharapkan Anda untuk menjawab pertanyaan yang terdapat didalam kuesioner ini dengan lengkap dan benar. Saya menjaga kerahasiaan data yang Anda isi. Silakan hubungi saya di nomor telpon +62xxx atau email ke [xxx@xxx.ac.id](mailto:xxx@xxx.ac.id) untuk Informasi lebih lanjut. Terima kasih atas kesediaan Anda dalam menjawab kuesioner ini.

Bagian responden

Name :

Email atau HP untuk validasi, tidak wajib diisi:

1. Jenis kelamin :
2. Perempuan
3. Laki-laki
4. Usia :
5. 15-20 tahun c. 31-35 tahun
6. 21-25 tahun d. > 35 tahun
7. 26-30 tahun
8. Pekerjaan :
9. Pelajar d. Ibu rumah tangga
10. Pegawai swasta e. Tidak bekerja
11. Pegawai negeri f. Lainnya……..
12. Pendapatan :
13. < Rp. 1.000.000
14. Rp. 1.000.000 – Rp.3.000.000
15. Rp.3.000.000 – Rp. 5.000.000
16. > Rp. 5.000.000
17. Tempat tinggal :
18. Urban
19. Rural

Mohon tandai/lingkari jawaban anda pada setiap pilihan yang tersedia dan pilih sesuai dengan keadaan/perasaan Anda yang sebenarnya. Anda hanya dapat memberikan satu jawaban di setiap pernyataan. Bacalah pertanyaan yang telah disediakan dengan seksama sebelum menjawab.

| Pernyataan | Sangat Setuju | Setuju | Tidak Setuju | Sangat Tidak Setuju |
| --- | --- | --- | --- | --- |
| Saya berniat membeli produk global/impor | 4 | 3 | 2 | 1 |
| Saya berniat membeli produk lokal/nasional | 4 | 3 | 2 | 1 |
| Saya membeli produk lokal karena desain produk memiliki ciri yang khas | 4 | 3 | 2 | 1 |
| Biaya yang dikeluarkan untuk memperoleh produk lokal terjangkau | 4 | 3 | 2 | 1 |
| Saya akan membeli produk global/impor dalam 12 bulan kedepan | 4 | 3 | 2 | 1 |
| Saya akan membeli produk lokal/nasional dalam 12 bulan kedepan | 4 | 3 | 2 | 1 |
